# Predictive role of PAR and LAR in refractory suppurative meningitis in infants

**DOI:** 10.1186/s12887-024-04898-6

**Published:** 2024-07-18

**Authors:** YaSong Gao, FangQi Hu

**Affiliations:** Department of Pediatrics, Anqing Municipal Hospital, Anqing, Anhui 246000 China

**Keywords:** Platelet/albumin, Lactate dehydrogenase/Albumin, Suppurative meningitis

## Abstract

**Background:**

Meningitis can be caused by a variety of pathogenic microorganisms, which can lead to higher mortality and disability rates. However, the clinical manifestations of suppurative meningitis are often atypical in infants and young children, which makes early clinical diagnosis difficult.PAR and LAR are considered as a novel inflammatory biomarker and have been applied in tumors, IgA nephropathy, sepsis.

**Objective:**

To investigate the application of platelet/albumin (PAR) and lactate dehydrogenase/albumin (LAR) in refractory suppurative meningitis in infants.

**Methods:**

The relevant clinical data of 107 children with suppurative meningitis were retrospectively analyzed, and were divided into common group (82 cases) and refractory group (25 cases) according to the severity of the disease according to the relevant clinical consensus. The relevant clinical data and laboratory examination of the children in the two groups were compared. The diagnostic value of PAR and LAR in children with refractory suppurative meningitis was analyzed and multivariate Logistic regression analysis was performed.

**Result:**

The PAR of children with suppurative meningitis in refractory group was lower than that in common group (*P* < 0.05), while LAR was higher than that in common group (*P* < 0.05). Meanwhile, multivariate Logistic regression analysis showed that LAR and cerebrospinal fluid glucose ≤ 1.5mmo/L were risk factors for poor prognosis (OR > 1, *P* < 0.05). PAR was a protective factor (OR < 1, *P* < 0.05).

**Conclusion:**

PAR and LAR can be used for early diagnosis of refractory suppurative meningitis in children as protective and risk factors, respectively.

## Introduction

Meningitis is a common central nervous system infection in children and infants, which can be caused by various factors, such as bacteria, viruses, fungi, tuberculosis, and parasites [1, 2]. It is also one of the top 10 causes of death for children under five [3]. The death rate is highest among children under 1 year of age (4.2%) [4].The disease has a global distribution, with approximately 2.8 million people suffering from meningitis each year, particularly in sub-Saharan Africa where the incidence is highest [5]. However, diagnosing suppurative meningitis in infants and young children can be challenging due to their clinical symptoms being atypical. Typical signs like neck stiffness and meningeal stimulation may not be present, making early diagnosis difficult. At the same time, due to the rapid development of refractory suppurative meningitis, the mortality and morbidity are higher than ordinary suppurative meningitis, and some children will have serious sequelae.Clinicians must rely on timely laboratory examinations and the targeted use of antibacterial drugs to identify and treat the condition. However, conventional laboratory indicators such as CRP, PCT, and WBC are not sensitive or specific enough for diagnosing suppurative meningitis, further complicating early identification.The level of lactate dehydrogenase (LDH) indicates the severity of cell injury and increases with infection [6]. Similarly, albumin (ALB) synthesized by liver cells not only reflects the body’s nutritional status but is also closely associated with inflammation, with its level decreasing as infection worsens [6]. Platelets (PLT) not only play a role in hemostasis and thrombosis but also serve as markers of systemic inflammation [7]. In recent years, to enhance the potential predictive value of each individual indicator, PCT and LDH have been combined with ALB to create new inflammatory markers called PAR and LAR. In light of this, we conducted a retrospective study to investigate the use of PAR and LAR in diagnosing refractory suppurative meningitis.

## Materials and methods

### Research objects and methods

#### Inclusion and exclusion criteria

In this study, we selected a total of 107 children aged 0–1 years old, comprising of 65 males and 42 females, who were admitted to Anqing Municipal Hospital, Anhui Province, China. These children were diagnosed with suppurative meningitis through lumbar puncture between July 2018 and July 2023. 

*In this study, the ***inclusion criteria***for* suppurative *meningitis were as follows*: **In newborns** [8, 9]:1) positive cerebrospinal fluid culture; 2) positive blood culture and increased white blood cell count in cerebrospinal fluid (> 21*10^*6*^cells/l); 3) negative cerebrospinal fluid culture or blood culture, but with clinical symptoms (such as fever, drowsiness, seizures, dystonia, bulging of the fontanelle, screaming) and increased white blood cell count in cerebrospinal fluid (> 21* 10^*6*^*cells/l). Among all newborns, the cerebrospinal fluid glucose concentration ranged from 0 ~ 11mmol/L, and the protein concentration ranged from 0.4 ~ 19.6 g/L. Children included in criteria 1 and 2 were defined as confirmed meningitis, and children with inclusion criteria 3 was defined as clinically diagnosed meningitis. ***In infants aged 29 days to 1 year** [10, 11]: *(1) sudden fever (> 38.5 ° C rectal or 38.0 ° C axial); (2) one of the following symptoms or signs appears: headache, neck stiffness, change in consciousness, or other meningeal signs;3)* any one of the following changes in cerebrospinal fluid: cloudy appearance, increased white blood cell count in cerebrospinal fluid (> 100*10^*6*^cells/l), increased CSF white blood cell count (10–100*10^*6*^*cells/l) with increased CSF protein (> 100 mg/dl) or decreased CSF glucose concentration (< 40 mg/dl);4) cerebrospinal fluid culture or blood culture positive, and with clinical symptoms. Children who meet inclusion criteria 1, 2, and 3 were defined as possible cases, while children who meet inclusion criteria 4 was defined as confirmed cases.*

#### Exclusion criteria

(1) Children who were treated with antibiotics or immunological agents before admission. (2) Children with inherited metabolic encephalopathy, bilirubin encephalopathy, congenital brain dysplasia, hydrocephalus, etc. (3) Children with meningitis caused by virus, fungus and tuberculosis infection. (4) Children with serious endocrine diseases, such as congenital hypothyroidism or adrenal hyperplasia. (5) Incomplete information.

According to Risk factors for poor prognosis in children with refractory purulent meningitis and the discharge criteria for the diagnosis of refractory suppurative meningitis [12]. the children with suppurative meningitis were divided into two groups: refractory group and common group. Patients with one or more of the following manifestations were classified into the dilemma treatment group: (1) Rapid progression of the disease, rapid onset of septic shock, respiratory and circulatory dysfunction, and even early death. (2) Repeated vomiting, convulsions, disturbance of consciousness, etc. (3) Serious complications such as cerebral infarction, cerebral hemorrhage, moderate to severe hydrocephalus, encephalomalacia, ependymitis, etc. (4) Those who did not meet the above criteria were classified into the general group. As this study was a retrospective study, the ethnic committee review board of Anqing Municipal Hospital in Anhui Province, China has waived the ethnics approval for the study, and the written informed consent of the parents or guardians of all infants was obtained for this study.

### Observation indicators

By referring to the electronic medical records of the children and recording their basic information, such as age, body temperature, respiration, heart rate and other indicators, and collecting the absolute platelet count, albumin, CRP, PCT, cerebrospinal fluid routine, biochemistry, cerebrospinal fluid culture, blood culture and length of stay of the children at admission, Where PCT level > 100ng/mL or < 0.02ng/mL is defined as 101ng/mL or 0.01ng/mL, respectively. PAR and LAR are calculated according to relevant data. PAR [13] is calculated as “absolute platelet count divided by serum albumin” and LAR [14] is calculated as “lactate dehydrogenase divided by serum albumin”.

### Statistical analysis

For normally distributed variables, continuous variables were represented by Mean ± SD, and for non-normally distributed variables, by median (quartile), and analyzed by independent sample t test or Mann-Whitney U test. Categorical variables are expressed as frequency (percentage), and comparisons between groups were made using Chi-square tests or Fisher exact probability methods. Variables with *P* < 0.05 in univariate logistic regression analysis were included in multivariate logistic regression analysis to identify risk factors for refractory purulent meningitis, as well as prespecified risk factors based on previously published literature [14], such as LAR.The significance of PAR and LAR in the diagnosis of severe suppurative meningitis was analyzed by the receiver operating characteristic (ROC) curve. Statistical analysis of all data was performed using IBM SPSS version 26.0. *P* < 0.05 was considered statistically significant.

## Results

### Study population characteristics

**Table 1:** Comparison of general data and laboratory examinations between the two groups

Variables	Suppurative meningitis		*P*
	Common group(*N* = 82)	Refractory group(*N* = 25)	
General condition			
Male [N (%)]	50(76.9)	15(23.1)	0.930
Age distribution [N (%)]			
0 ∼ 1 month	43(79.6)	11(20.4)	0.236^*^
1 ∼ 3 months	25(80.6)	6(19.4)	
3 ∼ 6 months	9(75.0)	3(25.0)	
6 months ∼ 1 year old	5(50.0)	5(50.0)	
Body temperature(℃)	38.70 ± 0.67	38.96 ± 0.91	0.120
Breaths (rate/minute)	41.23 ± 6.01	45.40 ± 10.74	0.074
Heart rate(bpm)	140.43 ± 13.67	146.48 ± 17.21	0.117
Length of stay (> 21 days) [N (%)]	15(57.7)	11(42.3)	0.009
Laboratory examination			
PCT(ng/ml)	0.50(0.17 ~ 2.24)	63.52(2.20 ~ 101)	0.000
CRP(mg/L)	39.58(7.60 ~ 89.40)	98.93(33.42 ~ 194.73)	0.000
WBC(10^9^/L)	10.03(7.87 ~ 13.50)	10.63(3.95 ~ 17.93)	0.491
PLT(10^9^/L)	360.13 ± 125.67	244.64 ± 161.76	0.000
ALB(g/L)	36.98 ± 3.84	33.42 ± 4.93	0.000
LDH(IU/L)	290(232.75 ~ 366)	448(295 ~ 642)	0.000
PAR(10^9^/g)	9.83 ± 3.33	6.30 ± 3.98	0.000
LAR(IU/L)	7.72(6.43 ~ 9.80)	15.31(7.67 ~ 19.51)	0.000
CSF WBC count ≥ 500*10^6^/L[N (%)]	23(62.2)	14(37.8)	0.010
CSF protein ≥ 2.5 g/L[N (%)]	9(40.9)	13(59.1)	0.000
CSF glucose ≤ 1.5mmol/L[N(%)]	11(40.7)	16(59.3)	0.000
Blood culture [N(%)]	9(56.3)	7(43.8)	0.241^*^
CSF culture [N(%)]	2(40.0)	3(60.0)	0.082^*^

Note: * Using Fisher exact probability method, PCT: procalcitonin, CRP: C-reactive protein, WBC: white blood cell count, PAR: platelets/albumin, LAR: lactate dehydrogenase/albumin

During the investigation period, a total of 107 infants met the diagnostic criteria for suppurative meningitis, among which 25 (23.36%) were refractory suppurative meningitis. In terms of gender distribution, the two groups were dominated by males, but there was no significant statistical difference between the sexes (*P* > 0.05). However, there was no statistical difference between the two groups at the four age groups (*P* > 0.05), and there was also no statistical difference between the two groups in body temperature, respiration, heart rate, WBC, cerebrospinal fluid culture, blood culture, etc. (*P* > 0.05). But the PCT、CRP、LDH、LAR in refractory group were significantly higher than those in common group (*P* < 0.001).PLT、PAR、ALB were significantly lower than those in common group (*P* < 0.001), and the proportion of CSF WBC count ≥ 500*10^6^/L in common group was higher than that in refractory group (*P* < 0.05). The proportion of CSF protein ≥ 2.5 g/L and CSF glucose ≤ 1.5mmol/L in refractory group was significantly higher than that in common group (*P* < 0.001).As shown in Table 1.

### ROC curve and predictive value of different indexes in the refractory group

**Fig. 1:**
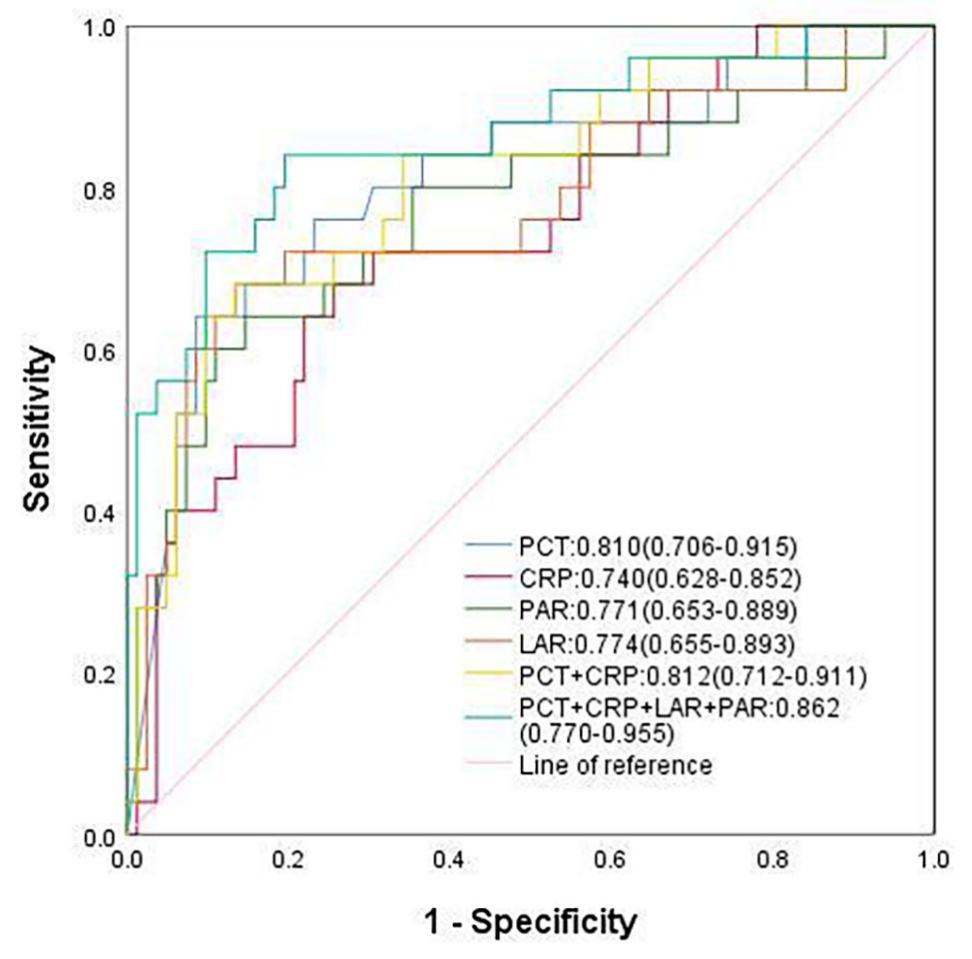
ROC curves of PCT, CRP, PAR, LAR, PCT + CRP, PCT + CRP + LAR + PAR

**Table 2:** Comparison of the predictive value of different indexes for children with suppurative meningitis in refractory group

Variables	Cut-off Values	AUC(95%Cl)	Sensitivity	Specificity	*P*
PCT	20.995	0.810(0.706–0.915)	0.640	0.915	<0.001
CRP	86.185	0.740(0.628–0.852)	0.680	0.744	<0.001
LAR	11.760	0.774(0.655–0.893)	0.680	0.866	<0.001
PAR	6.695	0.771(0.653–0.889)	0.640	0.854	<0.001
PCT + CRP		0.812(0.712–0.911)	0.680	0.866	<0.001
PCT + CRP + LAR + PAR		0.862(0.770–0.955)	0.840	0.805	<0.001

When performing receiver operating curve (ROC) analysis for PCT、CRP、PAR、 LAR、PCT + CRP、and PCT + CRP + LAR + PAR, We found that when using PCT alone as an inflammatory index in children with refractory suppurative meningitis, the optimal cutoff value was 20.995ng/ml, AUC(95%Cl) was 0.810 (0.706–0.915), and the sensitivity and specificity were 64% and 91.5%, respectively. The best cutoff value of CRP as an inflammatory index was 86.185 mg/L, and the sensitivity and specificity were 68% and 74.4%, respectively. When LAR and PAR were applied to refractory children with suppurative meningitis alone, the best cutoff value was 11.760IU/L and 6.695(10^9^/g), respectively. The sensitivity of LAR and PAR was 68%, 64%, and the specificity was 86.6% and 85.4%, respectively. The sensitivity of LAR was the same as that of CRP, but its specificity was higher than that of CRP. However, when we combined the relevant indicators (PCT + CRP + LAR + PAR), the sensitivity and specificity both reached more than 80%.As shown in Fig. 1; Table 2.

### Analysis of risk factors for prognosis of children in refractory group

**Table 3:** Variables associated with assignment for children with suppurative meningitis in the refractory group

Variables	variables name	Evaluation
Length of stay (> 21 days) [N (%)]	X_1_	*N* = 0, Y = 1
CSF WBC	X_2_	<500*10^6^/L = 0, ≥ 500*10^6^/L = 1
CSF protein	X_3_	<2.5 g/L = 0,≥2.5 g/L = 1
CSF glucose	X_4_	≤ 1.5mmol/L = 1, >1.5mmol/L = 0
Suppurative meningitis	Y	Common group = 0, Refractory group = 1

**Table 4:** Multivariate Logistic regression analysis of poor prognosis of suppurative meningitis in the severe group

Variables	Univariate		Multivariate	
	OR (95% CI)	*P*	OR (95% CI)	*P*
Sex(Male)	1.042(0.417–2.601)	0.930		
Age distribution				
0 ∼ 1 month	1			
1 ∼ 3 months	0.938(0.309–2.847)	0.910		
3 ∼ 6 months	1.303(0.301–5.638)	0.723		
6 months ∼ 1 year old	3.909(0.959–15.938)	0.057		
Body temperature	1.689(0.869–3.284)	0.122		
Breaths	1.073(1.011–1.139)	0.020	0.989(0.890–1.098)	0.830
Heart rate	1.027(0.997–1.058)	0.076		
Length of stay (> 21 days)	3.510(1.333–9.240)	0.011	0.490(0.072–3.356)	0.468
Laboratory examination				
PCT	1.031(1.018–1.045)	0.000	1.013(0.991–1.035)	0.239
CRP	1.010(1.004–1.017)	0.001	1.002(0.993–1.012)	0.664
PAR	0.740(0.632–0.867)	0.000	0.725(0.575–0.914)	0.007
LAR	1.184(1.078–1.301)	0.000	1.133(1.013–1.266)	0.028
CSF WBC count ≥ 500*10^6^/L	5.250(2.010-13.714)	0.001	1.604(0.311–8.275)	0.572
CSF protein ≥ 2.5 g/L	8.787(3.086–25.021)	0.000	6.161(0.822–46.180)	0.077
CSF glucose ≤ 1.5mmol/L	11.475(4.078–32.287)	0.000	6.052(1.044–35.067)	0.045

Adjusted for: Breaths、Length of stay、PCT、CRP、CSF WBC count ≥ 500*10^6^/L、CSF protein ≥ 2.5 g/L、CSF glucose ≤ 1.5mmol/L

We performed univariate analysis for age、sex、body temperature、respiration、heart rate、length of stay、PCT、CRP、PAR、LAR、CSF WBC count、CSF protein、CSF glucose. and multivariate analysis was performed for the relevant variables with *P* < 0.05 in the univariate analysis. LAR and CSF glucose concentration ≤ 1.5 mmol /L were found to be independent risk factors for refractory suppurative meningitis in children (OR > 1, *P* < 0.05), PAR was a protective factor for refractory suppurative meningitis in children (OR < 1, *P* < 0.05), as shown in Tables 3 and 4.

## Discussion

If children with bacterial meningitis do not receive timely and accurate treatment, it can result in serious neurological complications. Reports indicate that 10–30% of survivors experience long-term neurological damage, including hearing impairment and seizures [15]. These complications pose a significant threat to the life and health of children, especially infants and young children who have an immature blood-brain barrier. In addition, due to the immature immune system of newborns, the incidence is significantly higher than any other period, and its mortality is still high [16].In fact, the incidence of suppurative meningitis is significantly higher in infants and young children compared to older children, making it a leading cause of death and disability [4, 17, 18]. The rates of morbidity and mortality vary across different countries and regions, with developed countries showing significantly lower rates compared to developing countries [19].

LDH is an enzyme involved in cellular energy metabolism and can convert pyruvate into lactic acid [20]. It has been widely recognized as a predictor of disease severity, particularly in critically ill patients, as demonstrated by the study conducted by ZHANG D et al [21]. Previous research has shown that LDH is an independent prognostic factor for patients with sepsis [20]. Similarly, the severity of sepsis is correlated with the degree of thrombocytopenia [22, 23]. Infection-related thrombocytopenia has been attributed to platelet activation [24], which may occur through two mechanisms: (1) Platelet activation induced by pro-inflammatory cytokines, such as IL-6 and IL-8, in the highly inflammatory environment caused by infection [25]. (2) Interaction between platelets and infected endothelial cells, leading to platelet activation [26]. Previous studies have also found a correlation between hypoproteinemia in children with sepsis and prognosis [27].

Early identification and timely use of effective broad-spectrum antibiotics are crucial in the treatment of suppurative meningitis. Therefore, it is important for clinicians to quickly and accurately identify at-risk patients and identify readily available and cost-effective biomarkers. While WBC, PCT, and CRP are common inflammatory markers that still play a significant clinical role, they may not fully reflect the body’s infection status due to various factors. For instance, PCT levels increase physiologically after birth, reaching a peak within 24 h and gradually returning to normal levels within 48–72 h [28]. This limits the usefulness of PCT in diagnosing neonatal suppurative meningitis. PAR, a new inflammatory marker, has been applied in various conditions such as tumors, IgA nephropathy, and ankylosing spondylitis [13, 29, 30]. LAR has been used in sepsis and sepsis-related acute kidney injury [6, 31]. Both PAR and LAR combine two easily obtainable clinical indicators and are cost-effective, making them convenient for grassroots hospitals. In this study, we compared the levels of PCT, CRP, PAR, LAR, and combined indicators. We found that using PCT and CRP alone as inflammation markers resulted in a sensitivity of 64% and 68%, and a specificity of 91.5% and 74.4%, respectively. However, when combined, both sensitivity and specificity improved. The sensitivity of PAR and LAR was similar to that of PCT and CRP, while the specificity of PAR and LAR was higher than that of CRP but lower than that of PCT. Importantly, when PCT + CRP + LAR + PAR were combined, AUC was significantly increased, and its sensitivity and specificity were both above 80%. In addition, when PCR + CRP were combined, it also had higher sensitivity and specificity.

The results of this study indicate that refractory group biomarkers, including PCT, CRP, LAR, cerebrospinal fluid white blood cells ≥ 500*10^6^/L, and cerebrospinal fluid protein ≥ 2.5 g/L, were higher in the refractory group compared to the common group. On the other hand, PAR and cerebrospinal fluid sugar concentration ≤ 1.5mmol/L were lower in the refractory group than in the common group. Therefore, analyzing these indexes is crucial for early detection and diagnosis of refractory suppurative meningitis in children. Moreover, the multivariate Logistic regression analysis revealed that LAR and cerebrospinal fluid glucose ≤ 1.5mmo/L were risk factors for poor prognosis in children with refractory suppurative meningitis (OR > 1, *P* < 0.05), while PAR was a protective factor (OR < 1, *P* < 0.05). These findings align with previous studies [12].

However, our study also has some limitations. First, it is a retrospective, single-center study with a small sample size, which may subject these results to selection bias, and there are confounding factors.Secondly, PAR and LAR were not dynamically monitored and compared during hospitalization.In addition, due to the limited measurement level of laboratory measuring instruments, PCT levels > 100 ng/mL or < 0.02 ng/mL are designated as 101 ng/mL and 0.01 ng/mL, which may lead to bias.Finally, It should clear that this is an exploratory analysis and prospective studies are needed to draw conclusions on the performance of PAR and LAR.To confirm the usefulness of PAR and LAR in guiding prognosis and clinical treatment, we need larger clinical studies that use standardized methodologies.

## Conclusions

PAR and LAR not only aid in the diagnosis of refractory suppurative meningitis but also serve as risk and protective factors for poor prognosis in affected children. This suggests the importance of implementing active and effective measures to prevent the development of severe suppurative meningitis in these individuals.

## Data Availability

All data generated or analyzed during this study were included in this manuscript.
